# Supplementary figures and images for: Variation in phenotypes from a Bmp-Gata3 genetic pathway is modulated by Shh signaling
Source: PLoS Genet. 2021 May 25;17(5):e1009579. doi: 10.1371/journal.pgen.1009579 (PMC8184005; doi:10.1371/journal.pgen.1009579)

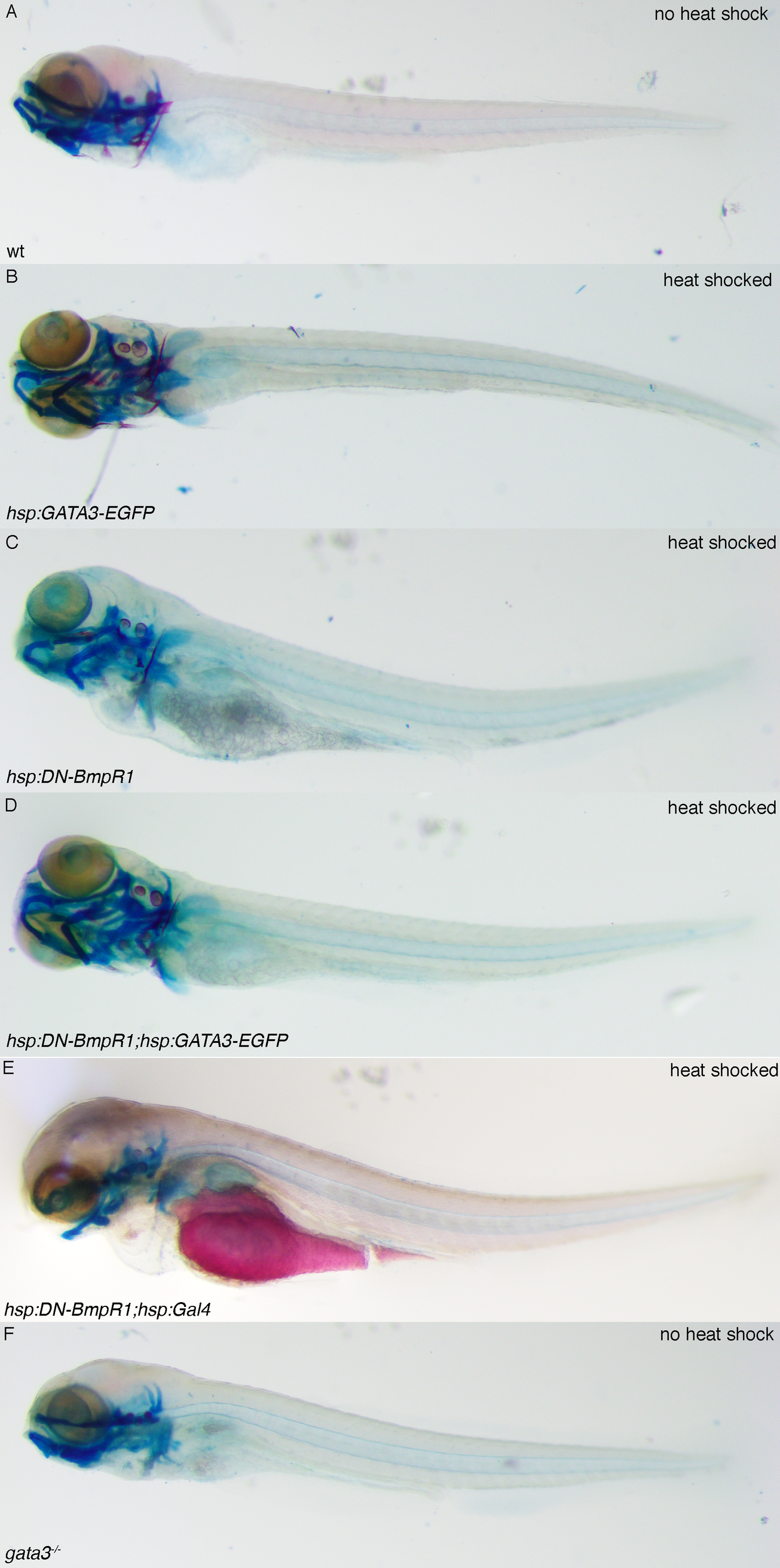

Supplement: S1 Fig — (A-D) Representative embryos of each genotype were imaged laterally with head to the left. Blue and red staining indicate cartilage and bone respectively (A) non-heat shocked wild type zebrafish. (B) Heat shocked wild type fish have normal morphology. (C) Heat shocked hsp:DN-Bmpr1a fish show some disruption to normal development, cardiac edema and alterations to the face. (D) Heat shocked hsp:DN-BmpR1a;hsp:GATA3-GFP fish appear normal. (E) Heat shocked hsp:DN-BmpR1a;hsp:Gal4 show similar disruptions to development as in C, providing genetic evidence that a double heatshock transgenic does not interfer with transgenic expression off of the heat shock promoter. (F) Overall, gata3 mutants have normal morphology. The image is of a gata3au42 mutant. (TIF) [file pgen.1009579.s001.tif]

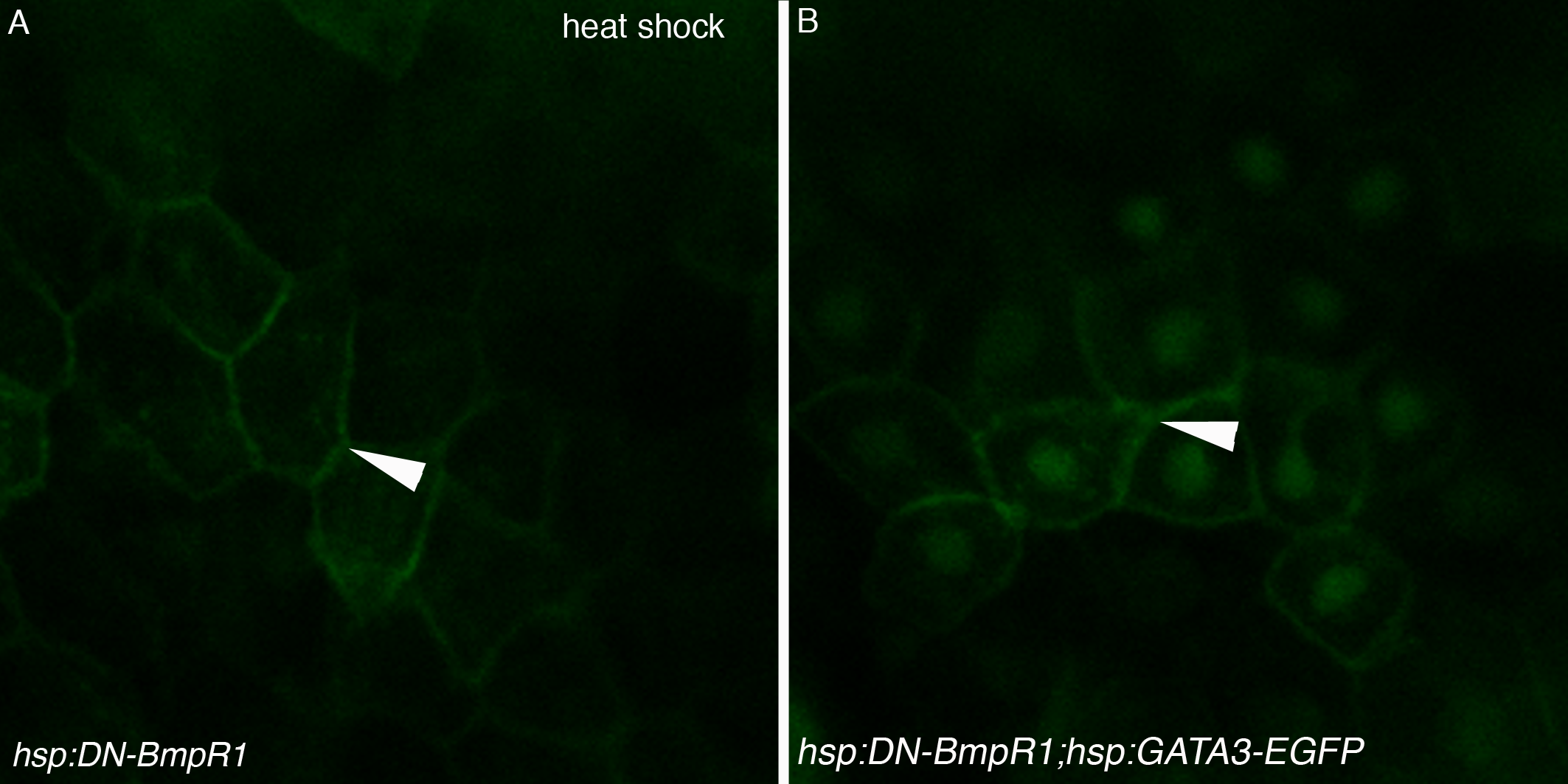

Supplement: S2 Fig — (A-B) Confocal single-z images of embryos at 30 hpf, embryos were heat shocked at 24 hpf. Representative images of (A) a hsp:DN-BmpR1a embryo and (B) a hsp:DN-BmpR1a;hsp:GATA3-EGFP embryo, arrows indicate cell membrane expression of DN-BmpR1-EGFP. Intensity values for hsp:DN-BmpR1a (n = 11, mean = 8663 arbitrary units (should be added for clarity), standard deviation = 1585, n = 11) were not statistically different from the intensity values for hsp:DN-BmpR1a;hsp:GATA3-EGFP (mean = 10583, standard deviation = 3463, n = 11), p-value = 0.1101 by t-test. (TIF) [file pgen.1009579.s002.tif]

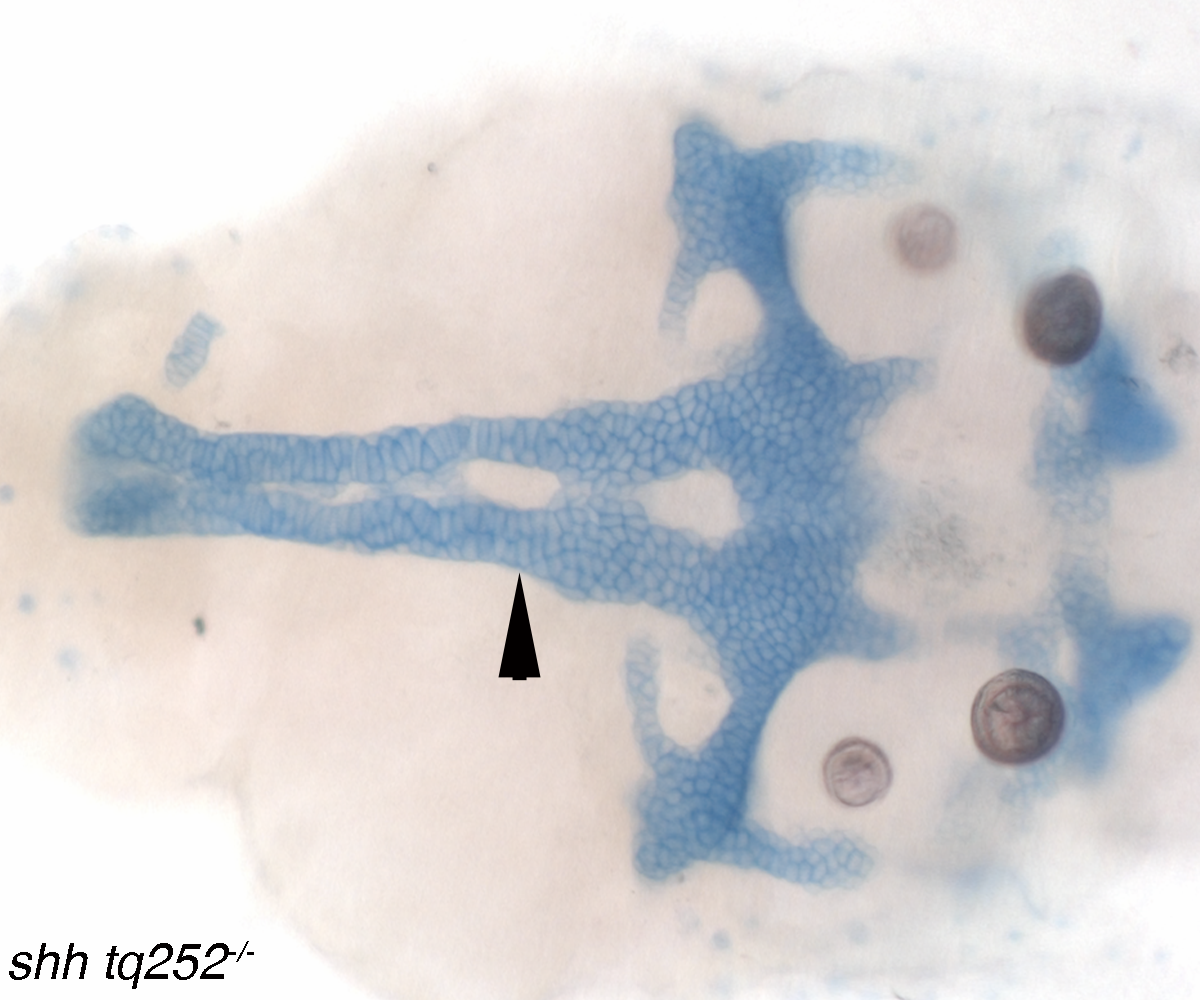

Supplement: S3 Fig — Flat mount of a 5dpf shhtq252/tq252 mutant neurocranium anterior to the left. Arrow is pointing to disrupted stacking of trabeculae cells. (TIF) [file pgen.1009579.s003.tif]

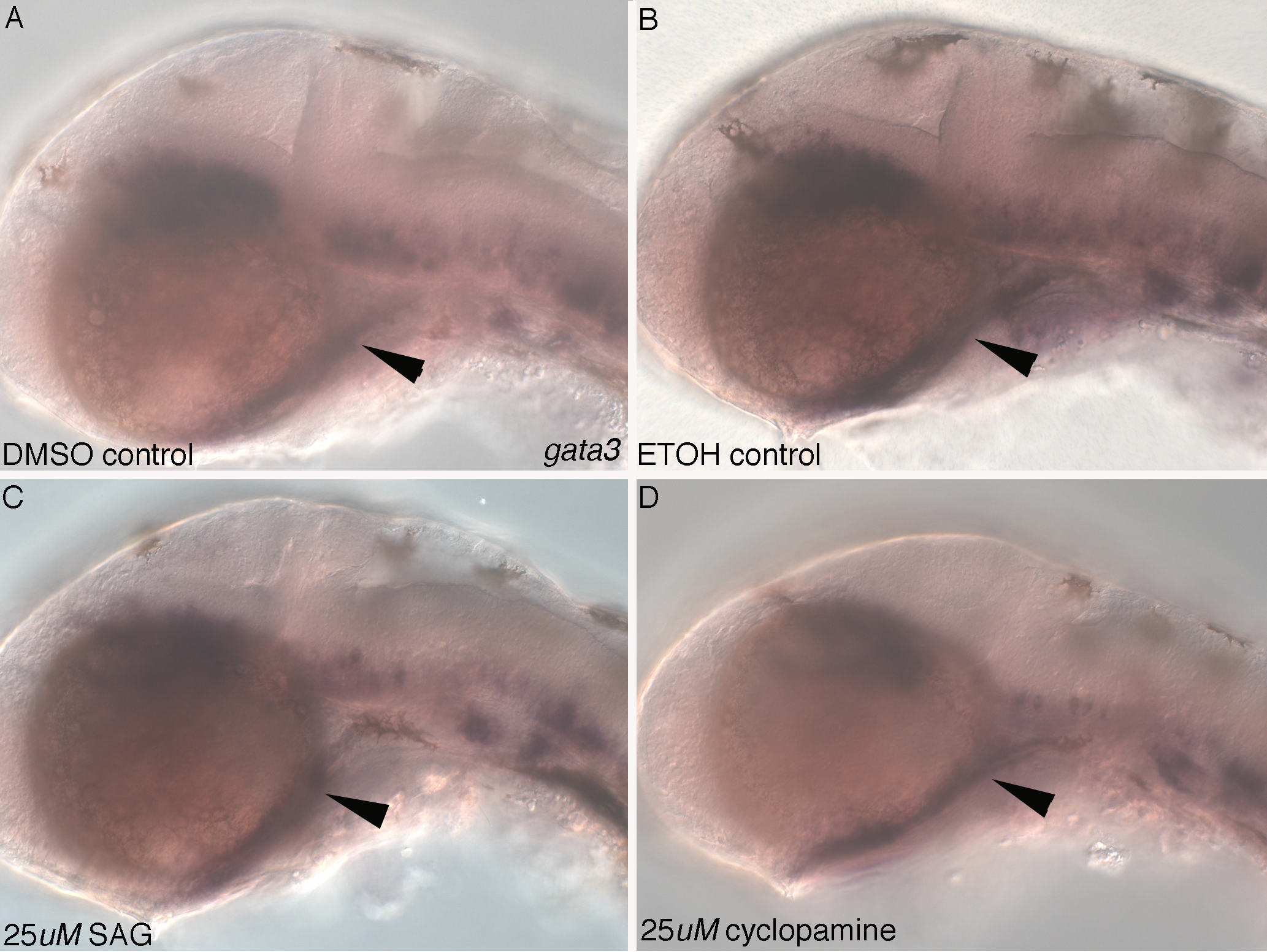

Supplement: S4 Fig — (A-D) Lateral views of embryos at 36 hpf showing normal expression of gata3 regardless of treatment. (A) Embryo treated with DMSO as a SAG treatment control. (B) Cyclopamine treatment control embryo treated with vehicle ETOH. (D) Embryo treated with 25uM SAG (C). 25 uM cyclopamine treated embryo. (TIF) [file pgen.1009579.s004.tif]

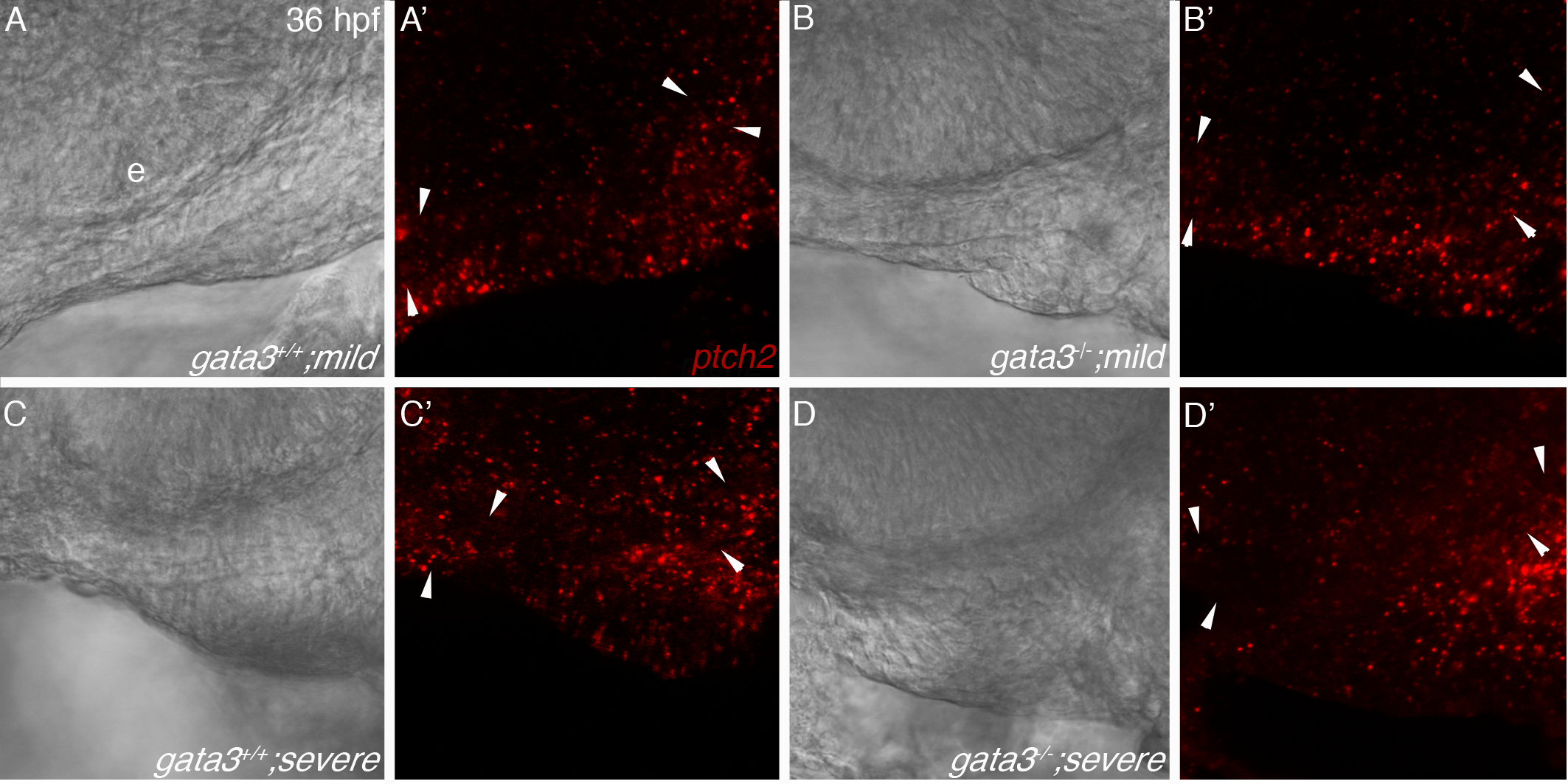

Supplement: S5 Fig — Single-z images of DIC and patched expression in the first pharyngeal arch. Arrows indicate maxillary neural crest. Eye is indicated by e. (TIF) [file pgen.1009579.s005.tif]
