# Supplementary material for: Variation in phenotypes from a Bmp-Gata3 genetic pathway is modulated by Shh signaling
Source: PLoS Genet. 2021 May 25;17(5):e1009579. doi: 10.1371/journal.pgen.1009579 (PMC8184005; doi:10.1371/journal.pgen.1009579)
Supplement: S6 Fig — (DOCX) [file pgen.1009579.s006.docx]

**Solutions to prepare**

0.2x SSCT (tween 0.01%)

PBST (tween 0.01%)

PBST +1%BSA

75% methanol in PBST

50% methanol in PBST

25% methanol in PBST

0.2M HCL in MEOH

5XSSC

1x Target Retrieval solution (comes as 10x in kit)

**Workflow**

**Part 1 Sample Preparation**

Collect zebrafish embryos at the desired developmental stages, and remove the chorions. Fix embryos in 4% paraformaldehyde fixation times are as follows: 4 cells to 8 hpf 4 hours, 12-20 hpf 1 hour, 24 hpf to 4 dpf 30 minutes. Do not agitate embryos, lay tubes on their sides during fixation.

**Dehydrate and Store the Embryos.**

1. Wash embryos in 1 ml 25% methanol in 1X PBST. Incubate at room temperature (RT) for 10 MIN.
2. Wash embryos in 1 ml 50% methanol in 1X PBST. Incubate at RT for 10 MIN.
3. Wash embryos in 1 ml 75% methanol in 1X PBST. Incubate at RT for 10 MIN.
4. Wash embryos in 1 ml 100% methanol. Incubate at RT for 10 MIN.

NOTE: Store the embryos in 100% methanol at –20^o^C for up to two months. Make sure the embryos do not dry out.

**Part 2 Sample Pretreatment**

**Rehydrate and Permeabilize the Embryos**.

1. For better penetration of solutions into the head remove yolk prior to beginning rehydration. We found this was essential for successful labeling of pharyngeal arches.
2. Transfer the desired number of embryos into one or more wells of a 24-well mesh insert that is inserted into a 24 well culture plate containing 2ml 100% MEOH. We added a second mesh to each well to prevent embryos from falling through the mesh.

NOTE: Transfer the embryos in 100% methanol to avoid the embryos sticking to the transfer pipette and mesh insert.

1. Transfer the mesh insert containing the embryos to a fresh 24 well plate containing 2 mL of 0.2 M HCl in 100% methanol. Incubate at RT for 30 MIN.
2. Transfer the insert containing the embryos to a fresh well plate with 2 mL of 75% methanol in 1X PBST. Incubate at RT for 10 MIN.
3. Transfer the insert containing the embryos to a fresh well plate with 2 mL of 50% methanol in 1X PBST. Incubate at RT for 10 MIN.
4. Transfer the insert containing the embryos to a fresh well plate with 2 mL of 25% methanol in 1X PBST. Incubate at RT for 10 MIN.
5. Transfer the insert containing the embryos to a fresh well plate with 2 mL of PBST + 1% BSA. Incubate at RT for 10 MIN. Bring water on hot plate to boiling and turn off. Float Target Retrieval solution in this water during next steps to bring to temp quicker.
6. Add 12-15 drops of Hydrogen Peroxide (provided in the kit) just make sure the embryos are covered incubate at RT for 10 Min.
7. Transfer the insert containing the embryos to a fresh well plate with 2 mL distilled water. Incubate at RT for 10 MIN (begin heating water to boiling on hot plate cover with foil).

**Apply RNAscope Target Retrieval.**

1. Pipette 2 ml of 1X Target Retrieval solution from 100°C water bath and place in well of culture dish.
2. Carefully transfer the insert containing the embryos into the heated 1X Target Retrieval solution. Wrap plate with lid on in two layers of foil Incubate at 100°C for 15 MIN by floating foil pack on water.
3. Immediately transfer the embryos to a fresh well plate containing 2 mL PBST + 1% BSA in a 24-well plate. Incubate for 1 MIN.
4. Transfer the insert containing the embryos to a fresh well plate, and wash with 100% methanol for 1 MIN.
5. Transfer the embryos into 1.5 mL microcentrifuge tubes, and carefully remove the 100% methanol.
6. Wash the embryos carefully by slowly adding 1 mL PBST + 1% BSA one drop at a time.

IMPORTANT! The embryos may stick to the side of the tube when PBST + 1% BSA is added. If this occurs, replace PBST + 1% BSA with 100% methanol and repeat steps 6 until embryos do not stick.

**Apply RNAscope Protease Plus.**

1. Carefully remove as much of the PBST + 1% BSA wash as possible without letting the embryos dry.
2. Add 300 μL of Protease Plus, and incubate at 40^o^C for 5–60 MIN depending on the age of the embryos (5−15 minutes for 24 hpf, 30 minutes for 48 hpf, and 60 minutes for 72 and 96 hpf). Float the tube horizontally in a water bath. During this wait time warm probe(s) by floating in the 40^o^C water bath.
3. Replace Protease Plus with 300 μL of Probe Diluent.

**Part 3 RNAscope Assay Probe Hybridization and Staining**

1. Remove Probe Diluent.
2. Add at 40^o^C diluted probe to embryos and incubate at 40^o^C floating in bath for 2 hours. It may help to preabsorb the probe for better signal to noise ratio. C2 and C3 probes are either diluted 1:50 in a C1 probe or Probe Dilutent.
3. Remove probe (this can be reused) Wash 2x 10 Min in 0.2X SSCT at RT (all 0.2X SSCT washes are 1ml).
4. Remove wash and add 1 ml of 4%PFA incubate for 10 Min at RT.
5. Remove PFA, Wash 2x 10 Min in 0.2X SSCT at RT.
6. Optional stopping point you can store the embryos overnight at RT in 5X SSC. If you do this Wash 2x 10 Min in 0.2X SSCT at RT before beginning the rest of the protocol.

**Hybridize AMP1.**

1. Remove wash and add 4 drops of RNAscope Multiplex FL v2 AMP1 to embryos and incubate at 40^o^C floating in bath for 30 Min.
2. Remove AMP1 wash 2x 10 Min in 0.2X SSCT at RT.

**Hybridize AMP2.**

1. Remove wash and add 4 drops of RNAscope Multiplex FL v2 AMP2 to embryos and incubate at 40^o^C floating in bath for 30 Min.
2. Remove AMP2 wash 2x 10 Min in 0.2X SSCT at RT.

**Hybridize AMP3.**

1. Remove wash and add 4 drops of RNAscope Multiplex FL v2 AMP3 to embryos and incubate at 40^o^C floating in bath for 15 Min.
2. Remove AMP3 wash 2x 10 Min in 0.2X SSCT at RT.
3. Prepare TSA Plus Opal fluorophores during this step. (1:2000 dilution in TSA buffer has worked well).

**Develop HRP-C1/C2/C3 Signals.**

NOTE: if you are using only C2 or C3 probe, choose the appropriate HRP channel (for example, RNAscope Multiplex FL v2 HRP-C2 or HRP-C3 instead of HRP-C1).

1. Remove wash and add 4 drops of RNAscope multiplex FL V2 HRP incubate for 15 Min 40^o^C
2. Remove HRP wash 2x 10 Min in 0.2X SSCT at RT.
3. Add diluted Opal fluorophore cover in foil and incubate at 40^o^C floating in bath for 30 Min.

NOTE: You can mix and match channels and fluorophores. For example, you may assign Opal 520 to channel C1and 570 to the C2 channel or vise-versa.

1. Remove Opal dye wash 2x 10 Min in 0.2X SSCT at RT.
2. Remove wash and add 4 drops RNAscope multiplex FL V2 HRP blocker, incubate at 40^o^C floating in bath for 15 Min.
3. Remove HRP blocker wash 2x 10 Min in 0.2X SSCT at RT.
4. Continue to develop the next channel in the same fashion just make sure to assign a different fluorophore to the second channel.
5. Remove wash and add 1 ml of PBST for 10 Min at RT.
6. Remove wash and add 1 ml of PBS keep covered in foil image immediately or store at 4 degrees O/N then image the next day.
7. Make sure to mount embryos in ProLong Glass Antifade Mountant for confocal imaging, as the dies are very unstable.
