# Supplementary material for: Variation in phenotypes from a Bmp-Gata3 genetic pathway is modulated by Shh signaling
Source: PLoS Genet. 2021 May 25;17(5):e1009579. doi: 10.1371/journal.pgen.1009579 (PMC8184005; doi:10.1371/journal.pgen.1009579)
Supplement: Attachment — Submitted filename: response to reviewers.docx [file pgen.1009579.s007.docx]

We wish to thank the reviewers and are pleased that they found our work to be well done and of interest. We have addressed the concerns of the reviewers and believe that the revised manuscript is substantially more impactful. We hope the reviewers agree that the revised manuscript is worthy of publication in PLoS Genetics.

Reviewer 1:

1) The description of parallel pathways is confusing.

Our new finding that Shh signaling is upregulated in the mild *gata3* mutants would suggest that the Bmp-Gata3 and Shh pathways are not entirely parallel. Because of this, we have removed this wording.

2) The reviewer’s main criticism was that stochastic differences were not demonstrated. The authors should reference their manuscript demonstrating Hsp90 alters the phenotypes of *gata3* mutants.

We fully agree, stochastic differences were only a potential mechanism by which variation could arise. While this still remains a possibility in general, our new FISH data demonstrate that the likely cause in our zebrafish is a compensatory upregulation of Shh signaling in the less expressive mutant. In the revised manuscript we have removed our discussion of stochastic differences and focused on this new and exciting finding.

We have also added the requested reference as part of our discussion of the possibility of other interactions modulating phenotypes (lines 546-551): “Our findings do not rule out the involvement of other pathways in modulating the phenotypes in *gata3* mutants. Indeed, we have previously demonstrated that altering Hsp90 activity can change the phenotypes in these mutants [13]. While the mechanism of this interaction is unknown, Hsp90 has a large number of client proteins involved in signaling [43]. However, our results clearly demonstrate that interactions between Shh and the Bmp-gata3 pathway modify the phenotypes generated in *gata3* mutants.”

3) Possible genetic compensation due to NMD.

In our experiments for this revision, we found that the presence and extent of apparent NMD varied. We no longer claim the allele is subject to NMD. However, we have added the potential for genetic compensation in CRISPR-generated mutants to the results section (lines 187-191): “In/del mutations, such as *au42*, can be associated with nonsense-mediated decay, which can result in genetic compensation [34]. However, the *b1075* allele is a mis-sense mutation, unlikely to undergo nonsense mediated decay. Thus, the similarity in phenotypes suggests a mechanism other than genetic compensation following nonsense mediated decay for the phenotypic variability.”

4) Test if *gata3* expression is lost in DN-BmpR1 embryos/if *gata3* mo enhances phenotypes in DN-BmpR1 fish.

We do find that *gata3* expression is lost in DN-BmpR1 embryos. These data have been incorporated into Fig. 6. Unfortunately, we were unable to interpret phenotypes of *gata3* mo-injected Dn-BmpR1 fish. Upon heat shock these fish develop severe, apparently nonspecific, defects throughout the body. While under normal circumstances this mo phenocopies the mutant, we have found non-specific interactions between morpholinos and environmental perturbations (ethanol exposure). We suspect that something similar is occurring in these fish. Based on this, we have toned down our interpretation of these results (lines 393-394): “While these findings do not rule out the possibility that other Bmp targets modulate the phenotype of *gata3* mutants, they suggest that *gata3* is a critical target.”

5) Analyze the expression of *gata3* in cyclopamine and SAG-treated embryos

We have performed these experiments and find no apparent alteration to *gata3* expression, supporting that *gata3* expression is independent of Shh signaling. These data are in S3 Fig.

6) Can you rule out mesoderm or posterior neural crest expression in 22 hpf embryos? How is maxillary neural crest expression verified in older embryos.

Based on morphology the expression at 22 hpf is likely to be endoderm, but the reviewer is correct that we cannot rule out other cell types. Since this population of cells is not the focus of the manuscript, we have changed our wording to “what is likely to be endoderm” (line 256). To verify that maxillary neural crest cells express *gata3*, we have performed fluorescent in situ hybridization to demonstrate overlap with *gata3* and pan-neural crest marker, *pdgfra*. These data can be found in revised Figure 3.

Minor comments

1) The phenotypic score between 2 and 1 is not obvious.

The reviewer is correct that this is a subtle difference that requires a trained eye. We feel that it is an important difference, however, in that the neural crest-derived cells are not fusing appropriately with the mesoderm-derived posterior neurocranium cells. In a phenotypic score 0, then, these two cell types fail to join altogether. We have clarified the precise nature of these scores in the revised manuscript to aid in the distinction (lines 194-201): “In zebrafish with the least severe phenotype the trabeculae fuses appropriately to the posterior neurocranium but is shortened and the cells within the trabeculae are inappropriately stacked but are in the same plane as and fused to the posterior neurocranium (Fig 2 B and F for our quantification below this is scored as a 2). We observed an intermediate phenotype in which the trabeculae are intact but have inappropriately stacked cells that fuse incorrectly to the ventral side of the posterior neurocranium so that the trabeculae and the posterior neurocranium are not in the same plane. (Fig 2 C, arrowhead, and G scored as a 1).”

2). Indicate inappropriate lateral commissure fusion with arrows.

These have been added to Fig 2.

3) Figures 6 and 7 should be combined.

We have combined these figures. These data are in Fig 6 in the revision.

4) Figures 9, 10, 11 and S1 should be combined.

We combined Figs 9 and 10 into Fig 8 of the revision. We have kept the data from Fig 11 in a separate Fig (Fig 9) because the haploinsufficiency observed in these fish is conceptually different from the modulation of mutant phenotypes in the other Figs. We have left S1 as a supplemental Figure because the neurocranial phenotype in *shha* mutants is published.

5). Use of au43 is confusing.

In the revised manuscript we have avoided use of the allele name and have instead used the transgenic name to avoid confusion.

Reviewer #2

Major comments

1) Fig 1 endogenous controls are needed for RT-PCR.

During our revision, we found variability in the extent of apparent nonsense mediated decay. Because of this we have removed the RT-PCR results.

2) Is there an interaction between severity of defects on the contralateral side?

In the revised manuscript, we have included these data in Table 1 and its relevant text (lines 224-229): “We further characterized the nature of the phenotypic variability by comparing phenotypes within larvae. We compared the score from the left and right trabeculae of 39 neurocrania (Table 1). Neurocrania with concordant scores (green boxes) are slightly less numerous (n=18) than those neurocrania that have discordant scores (blue and yellow boxes, n=21). Neurocrania scores differing by more than one (yellow boxes) are uncommon. This asymmetry fluctuates in its sidedness. However, the left side tends to have a less severe phenotype.”

3) Variation in sample size in Table 1.

It is true that the sample sizes vary, but we note that for all but one of the groups the samples are quite large. While the 18 hpf group is the smallest, at 12, it was included as a control because *gata3* is not expressed by maxillary neural crest cells at this time. We have elaborated on this in the revised manuscript (lines 286-287): “Embryos were heat shocked at times points ranging from 18 hpf, as a control for a time point prior to neural crest expression of *gata3*, to 48 hpf and then grown up to 4 dpf.”

4) Variability in phenotypes complicates the interpretation of the transplant data.

This is correct and there is no straightforward control for this. In the revised manuscript, we clarify this important point by stating that, across embryos, our transplants would target different sides of the embryo and that in all transplants the transplanted side was improved relative to the non-transplanted side. So while it’s true that no single transplant would definitively demonstrate a neural crest autonomous function of *gata3*, our findings across 10 embryos strongly supports this conclusion. In our revision we have also toned down the interpretation of the data (lines 308-313): “Of the 10 transplanted embryos six were chimeric on the left and four were chimeric on the right. While there is often phenotypic variability within an individual *gata3* mutant, we find that the phenotypic score on the transplanted side was always improved relative to the non-transplanted side. These results support a model in which neural crest cells require the function of *gata3* for proper trabeculae morphogenesis.”

5) qPCR for *gata3* could be useful.

We presume this statement is related to the in situ hybridization in the *smad5* mutants. It’s true that the in situ is not quantitative. However, there is a striking qualitative difference specific to the maxillary neural crest and our new data using *hsp:DN-BmpR1* (in Fig 6) further supports our previous finding. We feel that it is the spatial information provided by the in situ that is critical.

6) Fewer wild type embryos, relative to *gata3* heterozygotes, were treated with cyclopamine. A phenotypic quantification should be provided.

In these analyses we treated all the embryos from a cross of heterozygous fish. Therefore, the difference in numbers was unavoidable. We believe it is very unlikely that the difference in sample size obscured potential phenotypes in wild type embryos because this dose was initially tested in wild type embryos from the two different genetic backgrounds and we found that the dose did not disrupt the trabeculae. Furthermore, the dose of cyclopamine that we administered is well below that used to elicit defects in zebrafish at these time points. We have clarified this in the revised manuscript (lines 598-601): We used 12.5 uM cyclopamine to reduce Shh signaling to a level that doesn’t disrupt trabeculae development in either of the genetic backgrounds used in these analyses (n=26 WIK; n=28, EK), we note that typical zebrafish doses to disrupt craniofacial development at this age range between 50 and 100 uM.

Minor comments:

1) Referring to the anterior neurocranium as the palate is not agreed upon. It would be helpful to reference the structure as the anterior neurocranium.

In the revised manuscript, we have clarified this issue in the introduction (lines 92-94): “The zebrafish palate (also referred to as the anterior neurocranium) is comprised of an anterior, midline, ethmoid plate and the posterior bilateral trabeculae.”

2) Use of the word cleft is ill advised.

For simplicity, we have retained the use of the word cleft (and have removed the term clefting, as suggested). However, we have clarified the nature of the phenotype in the revised introduction (lines 162-165): “These phenotypes ranged from a severe truncation of the trabeculae, resulting in the failure of trabeculae to fuse to the mesoderm-derived posterior neurocranium (which we refer to as a cleft for simplicity) in one population, to mild cell rearrangements within the trabeculae, in a second population [13].”

3) There are numerous typos and inconsistencies.

These have been corrected.

Review 3

1) The *hsp:GATA3-GFP* transgenic causes expression everywhere, rescue could be due to expression in other tissues.

This is correct and in our submission we took care not to interpret that result as evidence for a neural crest requirement for *gata3*. However, the timing of the rescue matches when *gata3* is expressed by maxillary neural crest. Additionally, our transplant analyses demonstrate a neural crest autonomous requirement for *gata3*.

2) Images of whole embryos should be provided in the rescue experiments.

We have provided these images in S1 Fig. We find no major disruption to overall development. The only fish demonstrating a clear abnormal phenotype were the *hsp:DN-BmpR1* fish that displayed slight heart edema and facial defects as expected.

3) Many of the results are incremental.

Bmp signaling driving *gata3* expression in maxillary neural crest and the modulation of the resulting phenotype by Shh signaling has not been demonstrated. We hope that this and the new data quantifying a compensatory upregulation of Shh signaling adds to the novelty.

4) Overinterpretation of negative results: Potential effects of residual protein in *gata3* mutants and *gata3* knockdown in *smad5* heterozygotes.

As stated in the original submission, we sequenced cDNA from *gata3* mutants and demonstrated that the predicted frameshift is present in the mRNA. Thus, there is no evidence for residual protein in the mutants. The reviewer is correct though that the lack of exacerbation following *gata3* knockdown in a *smad5* heterozygote does not rule out a role of other Bmp targets. We have toned down our interpretation of these results (lines 393-394): “While these findings do not rule out the possibility that other Bmp targets modulate the phenotype of *gata3* mutants, they suggest that *gata3* is a critical target.”

5) Figures are minimalist. Figure 1 could be supplementary. Figures need better labels for those unfamiliar with the zebrafish skull.

We have retained Figure 1 in the manuscript. Related to the reviewer’s previous comment it is essential in understanding that there is no evidence for any residual function of *gata3*. Other figures have been combined as suggested by reviewer 1 and we have revised the figure labels.

6) How do the experiments with cyclopamine show a definitive role for Shh signaling?

New data provided in the manuscript demonstrate a compensatory upregulation of Shh signaling in the “mild” mutant background. We believe that this along with the cyclopamine data demonstrate that the upregulation of Shh signaling is functionally significant.

7) Other signals might influence variability in gata3 mutants.

The reviewer is absolutely correct and we do not mean to rule out other possible interactions. We have revised the discussion to reflect this (lines 546-551): “Our findings do not rule out the involvement of other pathways in modulating the phenotypes in *gata3* mutants. Indeed, we have previously demonstrated that altering Hsp90 activity can change the phenotypes in these mutants [13]. While the mechanism of this interaction is unknown, Hsp90 has a large number of client proteins involved in signaling [43]. However, our results clearly demonstrate that interactions between Shh and the Bmp-gata3 pathway modify the phenotypes generated in *gata3* mutants.”
