# Supplementary material for: Variation in phenotypes from a Bmp-Gata3 genetic pathway is modulated by Shh signaling
Source: PLoS Genet. 2021 May 25;17(5):e1009579. doi: 10.1371/journal.pgen.1009579 (PMC8184005; doi:10.1371/journal.pgen.1009579)
Supplement: Attachment — Submitted filename: response to reviewers.docx [file pgen.1009579.s008.docx]

We wish to thank the reviewers and are pleased that they found our work to be well done and of interest. We have addressed the remaining concerns of the reviewers and believe that the revised manuscript is substantially more impactful. We hope the reviewers agree that the revised manuscript is worthy of publication in PLoS Genetics.

Reviewer #1: The authors have largely addressed my initial concerns, except for one major concern and one more minor in nature.

Major: In Fig. S1, why does hsp:GATA3 also rescue the small eye defect and cardiac edema of hsp:DN-Bmpr1 mutants? There is a concern that rescue is possibly due to a non-specific effect of hsp promoter squelching. It is also very surprising that GATA3 can rescue all the defects of Bmp reduction as in different parts of the body presumably Bmp would have multiple targets. In other words, having two hsp transgenes may lead to "rescue" simply be causing reduced activity of the hsp:DN-Bmpr1a transgene due to competition with factors that bind to hsp promoters. Unless the authors have a good way of addressing this caveat (I can't think of one), this finding should be removed from the paper rather than just qualified with a comment such as "although we can't rule out....".

A non-specific competition model would suggest that any double hsp transgenic would show rescue. This is not the case from the literature (Kwon, Bhat, Sweet, Cornell and Riley, PLoS Genetics, 2010 and Bhat, Kwon and Riley, Developmental Biology, 2013 demonstrate additive effects in various combinations of *hsp:gata3, hsp:foxi1* and *hsp:tfap2a* double transgenics. This is an intriguing model though and we have directly tested the prediction with both genetic and imaging approaches. We have generated *hsp:DN-BmpR1a;hsp:Gal4* double transgenics. Given that Gal4 would have no function in these embryos, the nonspecific competition model would predict a rescue of the *hsp:DN-BmpR1a* phenotype. However, the phenotypes in these double transgenics mirror those of the single. These data have been incorporated into Supplemental Figure 1 Furthermore, given that the DN-BmpR1a and GATA3 gene products localize to different cell compartment (cell membrane and nucleus, respectively), we can directly quantify fluorescent intensity at the cell membrane. We visualized periderm cells given their large flat morphology, making it easy to distinguish cell membrane and nuclear labeling. We find no difference in intensity between *hsp:DN-BmpR1a* and *hsp:DN-BmpR1a;hsp:GATA3* transgenics. These data are in Supplemental Figure 2.

These other phenotypes are variable and we have not quantified them to determine if there is significant rescue. Because of this, we simply provide the necessary controls and do not comment on any potential rescue of other structures. We do note to the reviewer though, that *gata3* is expressed in the eye and while it is unclear if it is expressed in the heart, other Gata family members are expressed there.

Minor: The new data showing upregulation of ptch2 in mild gata3 mutants are compelling. While I understand the variability in expression and the need to carefully quantitate expression in just the maxillary regions, images of ptch2 in each condition for the whole head (lateral views) should be shown for perspective. For example, is the ptch2 upregulation in mild gata3 mutants just restricted to the maxillary domain?

We have included zoomed out images of the first pharyngeal arch in Supplemental Figure 5 to give readers an overall view of the first arch expression.

Reviewer #3: The revisions have addressed several of my comments and those of other reviewers, improving the paper, though the responses selectively leave out some points. My major remaining concern surrounds the rescue experiments and transplants, since both may rescue surrounding tissues. The transplants shown are not controls for cell type specificity since they only show sox10:mCherry positive donor NC cells, and not any other sox10-negative cells that were very likely included in the transplants. They also do not include evidence of contributions of transplanted cells to the skeleton on the rescued side (as requested in the initial review and not mentioned in the response). I realize this is difficult to address in with current restrictions, but it is pretty central to the interpretation. I also still worry that subtle phenotypes affecting posterior trabeculae are extremely variable depending not only on gene activity but fish health.

We apologize for misunderstanding the original concern of the reviewer. In revised Figure 5 we demonstrate that donor neural crest cells do populate the trabeculae. We transplanted from *ubi:Switch* embryos, which label all cells, into *gata3* morpholino-injected *sox10:mCherry* transgenics, labeling neural crest cells. While donor cells do contribute to the trabeculae, we cannot rule out the possibility that neural crest-derived cell types other than the chondrocytes of the trabeculae are the true cause of the rescue. In addition to the Figure, we have revised the text on lines 313-320:

“To determine if donor neural crest cells contribute to the rescued trabeculae, we transplanted cells from *ubi:Switch* (ubiquitously labeling cells green in the absence of Cre) into *sox10:mCherry* hosts injected with *gata3* morpholino. We find that these transplanted cells are capable of contributing to the trabeculae and that the trabeculae is rescued on the transplanted side (Fig 5. F-H). These results support a model in which neural crest cells require the function of *gata3* for proper trabeculae morphogenesis. However, it remains possible that *gata3* is not specifically required within the progenitors of the trabeculae, a genetic fate map of *gata3* descendants will aid in this understanding.”

Fish health: While we cannot rule out involvement of other factors, the fact that we see substantial variation of the trabeculae phenotype within a single fish strongly argues against fish health having a significant impact on the phenotype. We have added a photo of a whole *gata3* mutant larvae to Supplemental Figure 1, demonstrating that, overall, these mutants closely resemble wild-type larvae and do not have gross anatomical defects.
